# Measuring Integrated Care – The Quest for Disentangling a Gordian Knot

**DOI:** 10.5334/ijic.2525

**Published:** 2016-10-17

**Authors:** Roberto Nuño Solinís, K. Viktoria Stein

**Affiliations:** 1 Deusto Business School, University of Deusto, ES; 2 International Foundation for Integrated Care, GB

Transforming health care delivery to provide safer and more effective services that contribute to improving population health whilst containing costs is a significant challenge for health systems worldwide – a challenge that has been incorporated into the much cited Triple Aim Framework [1]. A key feature of transformation efforts has been to reduce the existing fragmentation both in the cure and care sectors, and support integration of systems, services, organisations, professionals and the wider communities depending on local context and needs.

A plethora of tools and concepts has emerged to potentially support this transformation and integration process e.g. [2]. Yet, the discussion on how to measure progress and success of integrated care has only recently started to pick up pace despite our understanding over many years that the lack of evidence on what works and what doesn’t in integrated care is partly due to the poor or non-existent evaluation and measurement of interventions e.g. [3].

From the history of innovation in integrated care it seems that people have shied away from investing in evaluation and measurement. Like the legend of the Gordian knot, the task is seemingly considered impossible – for example, amongst other reasons, researchers and practitioners alike comment that such interventions are too complex to understand, have too many stakeholders and perspectives involved, making it too difficult to attribute causality with too much of a time lag in observing any results. However, with the growing profile, interest and resources available to support integrated care initiatives, the demand for robust evidence and outcome measurement has become critical.

There are a number of ways in which the Gordian knot might be cut to advance our abilities to measure and evaluate integrated care. Before one embarks on the development of such measures and indicators, for example, one should be very clear on what purpose and perspective these should fulfil and take. Too often the evaluation of integrated care has been hampered by vague, unclear or non-existing goals and objectives. Defining the different purposes (e.g. accountability, informed decision making) and audiences (e.g. public, policymakers) for the evaluation will also help in breaking down the complexity of integrated care to a manageable subset. Another defining factor is the availability and access to (high-quality) data. As with public health interventions, there can be a significant timelag for when actual impact is measurable; so being clear on the objectives and their attainability over time must be considered.

If integrated care initiatives are to be truly able to provide the depth of evidence that we need then a measuring and monitoring framework should form an integral part of the overall transformational change strategy. Such a framework would need to be designed to support feedback on progress to populations, professionals, organisations and the system at large. Thus, approaches to measuring integrated care must be seen as an essential element of building a learning environment to support the Triple Aim.

In order to do so effectively, measuring the impact of integrated care from the perspective of patients and service users (in terms of their care experiences as well as their health outcomes) needs to start on equal terms with measuring impacts from the systems’ or organizational perspectives. Indeed, measuring people’s experience of care has become the new trend in healthcare management. In many places, traditional patient satisfaction surveys are being replaced with care experience surveys with minimal changes in content, yet these questionnaires continue to focus on episodic care, adopt a fragmented focus with separate questions for doctors, nurses and other providers, and do not consider IT innovations that are changing the traditional interactions between healthcare staff and patients.

Fortunately, in the field of integrated care, the measurement of people’s experience with integrated chronic care is rich in terms of new tools (or updates of existing ones) that are challenging the standard approaches. In fact this area of knowledge has been called the ‘next frontier in health care delivery’ [4]. Although, a systematic review performed in 2009 by Vrijhoef et al. [5] only identified one appropriate patients’ survey for measuring quality of integrated chronic care: the PACIC (Patient assessment of chronic illness care) based on Wagner’s Chronic Care Model [6], in the last 6 years new tools have been developed like:

– PACIC+, the PACIC extended with six additional multidisciplinary team functioning items [7]
– Patient Perceptions of Integrated Care [8]
– The scales developed by Walker et al. [9]

Recently published papers in IJIC have also set out the results of new approaches seeking to develop tools for measuring the experience of patients with chronic illness care such as the IEXPAC [10], developed in Spain, or adaptations of existing surveys such as the Dutch validation of the Patient Perceptions of Integrated Care [11].

This growth in care experience measures, instruments and tools tends to confirm the idea that health systems must ask people directly in order to understand their experiences within a “system of care” if they truly want to deliver people-centered care in a seamless and coordinated way. This issue is very important, because in many political agendas integrated care remains a synonym for IT integration, organizational mergers and other structural initiatives without paying enough attention to the outcomes of integration, the patient experience being key.

Although, the relationship between reported care experiences and quality of care is not consistent in the literature, these developing tools are seeking to make the link between care experiences and the quality of key integration processes – for example, intra- and inter-team coordination, continuity of care, involvement in decision-making, self-management support, continuity of information, and so on. These co-ordination mechanisms are essential aspects to measure and should be routinely gathered and analysed in systems that are developing care integration strategies.

Measuring integrated care on all levels and taking into account the different perspectives is certainly a complex task but not unattainable. It needs a clear strategy, measurable objectives and outcomes, as well as high-quality data, which are routinely fed back into the system. In order to achieve this, many initiatives are currently underway from the WHO, the OECD, the European Union, and national and regional governments to develop new frameworks and identify indicators. e.g. [2,12,13] Currently, these are still based on existing health system performance assessment tools and indicators.

An important next step will thus be to identify the gaps between what is already measured and relevant for integrated care and which aspects of integrated care are not yet mirrored in the measurement. As with all other aspects of integrated care, introducing adequate performance measurement can be a costly undertaking, which needs money, time and expertise specifically allocated to it. Equally, for performance measurement to be effective it needs to be aligned with other levers for system improvement such as financing, accountability arrangements and regulations.

To conclude, and reiterating what has been said before, the development of measuring and monitoring frameworks for integrated care is essential and must be part of a care system’s overall improvement or transformational change strategy. This implies fresh thinking on traditional approaches to health systems performance. As with the legend of the Gordian knot, seemingly intractable problems like measuring integrated care will only be solved by ‘thinking outside the box’.
